# Intraventricular Neurilemmoma (Schwannoma): Shall GFAP Immunostaining Be Regarded as a Histogenetical Tag or as a Mere Histomimetical Trait?

**DOI:** 10.1155/2016/2494175

**Published:** 2016-06-30

**Authors:** Miguel Fdo. Salazar, Martha Lilia Tena Suck, Daniel Rembao Bojórquez, Citlaltepetl Salinas Lara

**Affiliations:** ^1^Pathology Unit, Neuropathology Service, Mexico General Hospital, Cuauhtémoc, 06726 Mexico City, DF, Mexico; ^2^Department of Neuropathology, National Institute of Neurology & Neurosurgery “Manuel Velasco Suárez”, Tlalpan, 14269 Mexico City, DF, Mexico

## Abstract

Neurilemmomas are benign neoplasms presumedly derived from Schwann cells which rarely originate within the central nervous system. Moreover, their intraventricular location has been seldom noticed with less than 30 cases reported worldwide. Here, we add another case study to the record as well as the fifth one in Latin American population. A 16-year-old boy without significant past clinical data debuted with headache and progressive left eye blindness during six months. Neuroimaging scans showed a bulky, multiloculated, intraventricular tumour emerging from the posterior horn of the left lateral ventricle. Microscopically, the lesion put on view the classical schwannian histology: spindle cells arranged in both compact and loosely textured areas. Verocay bodies were not present but vessel hyalinisation, pericellular reticulin, and senescent atypia were observed. The immunoperoxidase reactions were also consistent with neurilemmal differentiation; however, glial fibrillary acidic protein expression was widespread and unexpectedly seen. Traditionally conceived as “*nerve sheath tumours*” the dual immunophenotype herein demonstrated points to a different histogenetical pathway other than sheer Schwann cell derivation. As previously advised by some authors, neoplastic transformation from a multipotent stem cell may explain the occasional finding of these tumours in unconventional intracranial compartments.

## 1. Introduction

Canonically assumed to sprout from tissue encasing nerve extensions, the so-called* nerve sheath*, neurilemmomas prove to show a Schwann cell phenotype, hence their more popular designation which springs their name:* schwannomas*. The vast majority of neurilemmomas grow outside the central nervous system or are associated with spinal nerve roots [1]. Intracranial examples, on the other hand, are usually attached to cranial nerves, most commonly VIII, V, VII, and XII [1, 2], but have been also seldom noticed in intraaxial and intraventricular locations [1]. The latter represents the most uncommon topography, with nearly thirty case reports written worldwide [3–29]. Here, we present the 29th case as well as the fifth one registered in Latin America (Table 1).

## 2. Case Report

**Figure 1:**
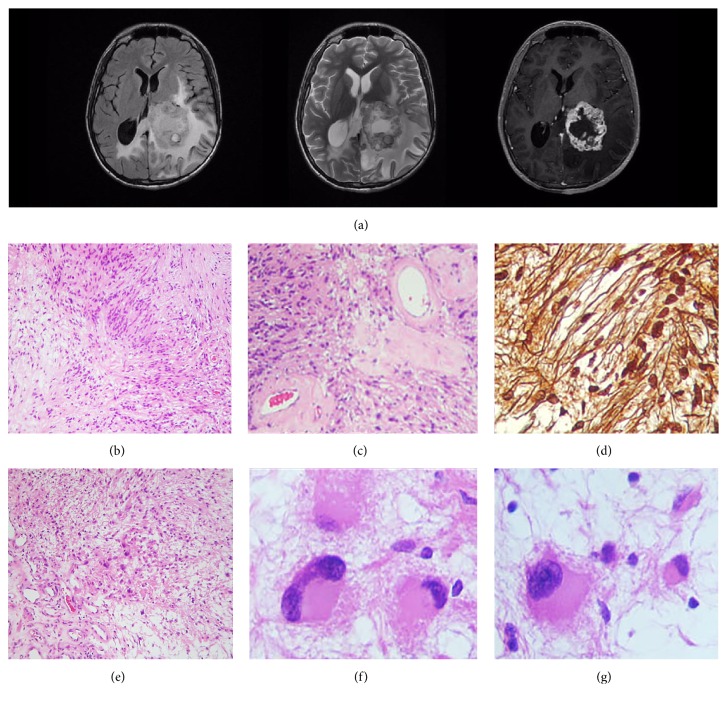
Magnetic resonance imaging scans/histopathological findings. (a) Postcontrast T_1_ (*right*) and T_2_ weighted (*center*) sequences demonstrate a cystic, avidly enhancing mass, while the fluid-attenuated inversion recovery (FLAIR) sequence (*left*) shows enlargement of the* cornu occipitale sinister* with surrounding white matter oedema, consistent with ventricular entrapment; this might as well explain the blurred vision due to affection of the geniculocalcarine tract. (b) Transition zone, in a spindle cell neoplastic population, from an Antony A pattern (*left*) to an Antony B area (*right*). H&E. (c) High magnification photomicrograph showing intratumoural hyalinised vessels. H&E. (d) Reticular fibers stain. A pericellular reticulin frame, trademark of neurilemmal phenotype, is noticeable. (e) Antony B field with a small population of neoplastic, plump, gemistocyte-like cells. H&E. ((f), (g)) High magnification photomicrographs of the cells shown in (e): voluminous cells with eccentric nuclei and senescent atypia are evident.

**Figure 2:**
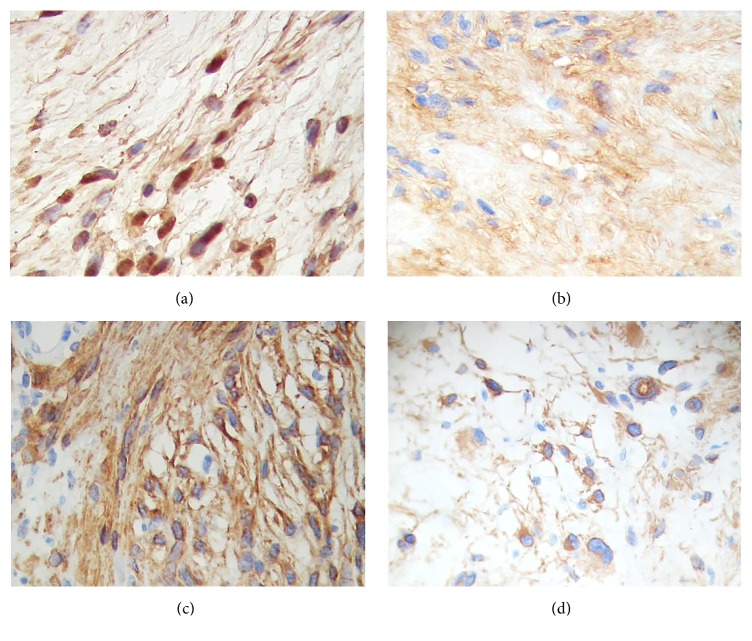
Immunohistochemistry panel. (a) PS100. (b) Collagen IV. ((c), (d)) Glial fibrillary acidic protein (GFAP) in an Antony A area (c) and in Antony B gemistocyte-like cells (d).

A 16-year-old boy without any pathological, genetic, or familial history of significance suffered from headaches and intermittent blurry vision during six months; he got alarmed when his left eye vision was completely lost and presented to an urgent care facility. Magnetic resonance imaging scans showed an intraventricular, irregularly nodular, space-occupying lesion emerging from the posterior horn of the left lateral ventricle (Figure 1(a)). A gray-yellow tumour attached to the* glomus choroideum* was excised* in integrum* by the surgeon; histopathological examination disclosed a biphasic neoplasm laid out in densely packed fascicles of spindle cells with elongated nuclei (Antoni A pattern) as well as loosely clustered, hypocellular areas in a myxoid background (Antoni B pattern) (Figure 1(b)). Although Verocay bodies were not conspicuous, some blood vessels had hyalinised walls (Figure 1(c)) while a reticulin lattice enveloped every single cell (Figure 1(d)). Senescent changes were also present, with isolated cells in the Antoni B areas displaying a round, voluminous, eosinophilic cytoplasm with a displaced atypical nucleus; some of them even resembled a gemistocytic morphophenotype (Figures 1(e)–1(g)). The immunohistochemistry panel confirmed the diagnosis of neurilemmoma: PS100^(+)^, collagen IV^(+)^, and vimentin^(+)^ (Figures 2(a) and 2(b)), but also unveiled a diffuse labelling for glial fibrillary acidic protein (GFAP) in both Antony patterns (Figures 2(c) and 2(d)). Thus, according to the aforementioned traits, the case was regarded as an* intraventricular neurilemmoma*.

## 3. Discussion

It has been estimated that neurilemmomas account for approximately 10% of intracranial tumours, the majority of them (~85%) arising at the cerebellopontine angle [1, 3, 4]. Conversely, the intraventricular location is very unusual; for instance, in the series published by Luo et al. [4] about 18 non-cranial nerve related neurilemmomas—the largest up to date—only one (0.055%) was intraventricular. Our surveillance revealed just 28 cases documented from 1965 to the present (Table 1). Moreover, none of them has been associated with either neurofibromatosis or any other phakomatoses. It is worth mentioning the existence of three more cases labeled as* malignant schwannomas* [30–32]; however, we decided to cast them aside as they correspond in fact to neurogenic sarcomas, that is, malignant peripheral nerve sheath tumours (MPNST), and, thus, are not true neurilemmomas.

Etiopathogenesis of such a neoplasm within the cerebral ventricles is perplexing and, hence, several hypotheses have been introduced [3–7]: (1)* growth from autonomic nerve cells inherent to choroid plexus or from nervi vasorum*, a premise based on the identification of the former by Benedikt in 1874 and confirmed by Stöhr in 1922 [33, 34] (also, the apparent attachment to* choroid plexus* supports this conjecture), (2)* development from displaced neural crest cells* which, in turn, may give rise to ectopic Schwann cells, and (3)* neoplastic transformation of multipotent stem cells*. Although no sole speculation may be sufficient to explain every single case—as different, nonexcluding mechanisms shall be involved—recent advances in the recognition of the so-called “neural stem cells” make the third assumption particularly attractive. Indeed, Doetsch et al. [35] confirmed the existence of such a lineage placed in the subventricular zone; furthermore, they seem to bear the cardinal phenotypical traits of astrocytes. Interestingly, it is well known that, in contrast to their peripheral counterpart, non-cranial nerve related neurilemmomas are more prone to diffusely express GFAP [3]. Unfortunately, just a couple of reports emphasise this difference and demonstrate it in their photographs [5–7]. In this regard, we share the opinion of Luo et al. [4] stating that at least this subset of neurilemmomas arises from neoplastic transformation of subventricular pluripotent stem cells, hence preserving parental GFAP^(+)^ immunophenotype which may in fact represent some kind of* histogenetical imprinting*.

The clinical presentation of intraventricular neurilemmomas generally involves headache, nausea, and vomiting; however, brachial-crural hemiparesis, seizures, vertigo, and visual symptoms such as homonymous hemianopsia and transient scintillating scotomas have also been reported [3–7]. On the other hand, common neuroradiological features include a heterogeneously enhancing, predominantly solid mass with intralesional cysts and overstressed peritumoural oedema [3–7].

Differential diagnoses in our setting include cystic ventricular-extending astrocytoma, cystic meningioma, ependymoma, choroid plexus papilloma or carcinoma, haemangioblastoma, and metastatic lesions [3, 5–7]. Some morphological traits such as absence of papillary stalks lined by a single layer of uniform cuboidal/cylindrical epithelial cells, nonattendance of plump lipid-laden multivacuolated stromal cells, or lack of foreign carcinomatous/sarcomatous invading cells easily discard the latter by simple light microscopy examination. Conversely, ependymoma, meningioma, and astrocytoma may demand further analysis by means of immunohistochemical evaluation. In this regard, employment of reagents such as PS100, GFAP, epithelial membrane antigen (EMA), cytokeratin-18 (CK-18), progesterone receptors (PR), and vimentin should be useful to guide diagnosis: for instance, diffuse EMA, CK18, PR, and vimentin immunolabeling is akin to meningiomas while PS100, GFAP, and ring/dot-like EMA positivity is seen in ependymomas. Of particular interest and difficulty to exclude in our case are pilocytic astrocytoma and tanycytic ependymoma, as both of them share a long spindle cell morphology as well as PS100, GFAP, and vimentin immunostaining; none of them, however, bear the distinctive reticulin lattice of neurilemmomas which corresponds to the continuous basal lamina coating every single neoplastic Schwann cell. Moreover, in spite of PS100 common employment as a key immunohistochemical marker for schwannian neoplasms, it is not essentially needed, as sole H&E morphology is diagnostic (Antoni growth patterns, Verocay bodies, lipid-laden cells, and thick-walled hyalinised vasculature). Curiously, it is well known that intracranial neurilemmomas usually lack formation of Verocay figures [1].

Finally, does GFAP positivity in neurilemmomas solve the puzzling issue of etiopathogenesis? Not necessarily, as this hypothesis is based on casual observations rather than explicit experimental data. Nevertheless, we think it is firmly revealing. On the other hand, there might be the possibility for the neoplastic cell to adopt new lineage clusters, a phenomenon we have coined as* histomimesis*, and, thus, it would not reliably reflect its native roots. Whether it is one way or the other is a field of inquiry.

In spite of their exceptional rarity, neurilemmal neoplasms are a diagnosis to consider during the evaluation of intraventricular tumours, making the recognition of this benign and potentially curable lesion of obvious importance. Hence, we add a new case of intraventricular neurilemmoma, the twenty-ninth one to the global registry as well as the fifth known to Latin American population.

## Figures and Tables

**Table 1:** Intraventricular neurilemmomas case list.

Case number	Year	Author [reference] (country)	Age/gender	Location
1	1965	David et al. [8] (France)	15♂	Lateral ventricle
2	1975	Ghatak et al. [9] (USA)	63♀	Lateral ventricle
3	1975	Van Rensburg et al. [10] (South Africa)	21♂	Lateral ventricle *(cornu temporale)*
4	1988	Pimentel et al. [11] (Portugal)	8♂	Lateral ventricle *(right)*
5	1990	Ost and Meyer [12] (USA)	44♂	Lateral ventricle *(left; cornu occipitale)*
6	1990	Redekop et al. [13] (Canada)	7♂	4th ventricle
7	1993	Weiner et al. [14] (USA)	61♂	4th ventricle
8	1993	Weiner et al. [14] (USA)	78♀	4th ventricle
9	2001	Barbosa et al. [15] (Portugal)	13♀	Lateral ventricle (a*trium)*
10	2002	Estrada et al. [16] (Mexico)	36♀	4th ventricle
11	2003	Erdogan et al. [17] (Turkey)	21♂	Lateral ventricle
12	2004	Dow et al. [18] (UK)	16♂	Lateral ventricle *(right; *a*trium)*
13	2006	Messing-Jünger et al. [19] (Germany)	21♀	3rd ventricle
14	2007	Lévêque et al. [20] (Belgium)	16♂	Lateral ventricle *(right)*
15	2008	Benedict et al. [21] (USA)	15♂	Lateral ventricle *(right; cornu occipitale)*
16	2009	Oertel et al. [22] (Germany)	71♀	4th ventricle (^*∗*^ *cellular variant)*
17	2009	De Vasconcellos et al. [7] (Brazil)	21♀	Lateral ventricle *(left; atrium)*
18	2010	Martin et al. [23] (Czech Republic)	70♀	3rd ventricle
19	2011	Hodges et al. [6] (USA)	69♂	4th ventricle
20	2012	Kachhara et al. [24] (India)	30♂	4th ventricle
21	2013	Chen et al. [25] (China)	53♀	4th ventricle
22	2013	Luo et al. [4] (China)	24♂	Lateral ventricle *(righ*t)
23	2013	Jaimovich et al. [26] (Argentina)	16♂	Lateral ventricle *(right; cornu occipitale)*
24	2013	Alberione et al. [27] (Argentina)	41♀	Lateral ventricle *(right; cornu occipitale)*
25	2015	Li et al. [28] (China)	23♂	3rd ventricle
26	2015	Glikstein et al. [29] (Canada)	34♂	Lateral ventricle *(left)*
27	2015	Currán-Meléndez et al. [3] (USA)	20♂	Lateral ventricle *(right; *a*trium)*
28	2016	Abdolhosseinpour et al. [5] (Iran)	9♂	Lateral ventricle *(left; atrium)*
29	2016	Present Case (Mexico)	16♂	Lateral ventricle *(left; cornu occipitale)*

*Statistical summary*				
Adults: 19 (65.5%) ∣ paediatric: 10 (34.5%) ∣ mean age: 32 y				
Male: 19 (65.5%) ∣ female 10 (34.5%)				
Age range: 7 y to 78 y				
Lateral ventricles: 18 (62%) ∣ 3rd ventricle: 3 (10.3%) ∣ 4th ventricle: 8 (27.6%)				

♂: male, ♀: female, y: years.

^*∗*^Special histopathological variant.
